# Supplementary material for: Splice-Junction-Based Mapping of Alternative Isoforms in the Human Proteome
Source: Cell Rep. Author manuscript; Available in PMC 2020 Jan 15. (PMC6961840; doi:10.1016/j.celrep.2019.11.026)
Supplement: 3 [file NIHMS1546469-supplement-3.zip › DF2/PXD0010154/AdrenalGland-201-Q15149-MEEEEMQTVQQEQLLQETQALQQSFLSEK.pdf]

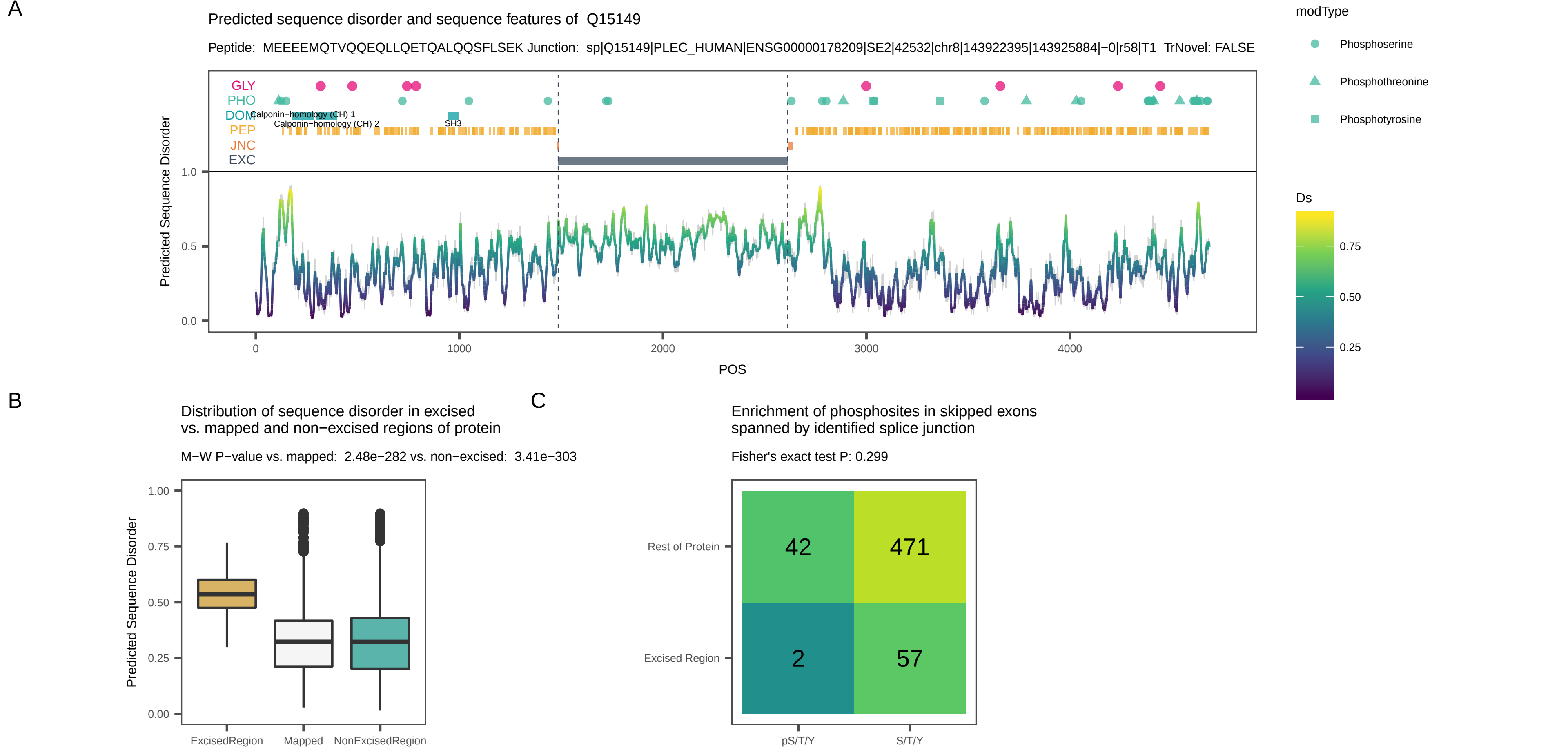
A
Predicted sequence disorder and sequence features of Q15149
Peptide: MEEEEMQTVQQEQLLQETQALQQSFLSEK Junction: sp|Q15149|PLEC_HUMAN|ENSG00000178209|SE2|42532|chr8|143922395|143925884|−0|r58|T1 TrNovel: FALSE
GLY
PHO
DOM
PEP
JNC
EXC
Calponin−homology (CH) 1
Calponin−homology (CH) 2
SH3
Predicted Sequence Disorder
1.0
0.5
0.0
0
1000
2000
3000
4000
POS
modType
Phosphoserine
Phosphothreonine
Phosphotyrosine
Ds
0.75
0.50
0.25
B
Distribution of sequence disorder in excised vs. mapped and non−excised regions of protein
M−W P−value vs. mapped: 2.48e−282 vs. non−excised: 3.41e−303
Predicted Sequence Disorder
1.00
0.75
0.50
0.25
0.00
ExcisedRegion
Mapped
NonExcisedRegion
C
Enrichment of phosphosites in skipped exons spanned by identified splice junction
Fisher's exact test P: 0.299
Rest of Protein
Excised Region
42
471
2
57
pS/T/Y
S/T/Y
